# Reconfigurable application-specific photonic integrated circuit for solving partial differential equations

**DOI:** 10.1515/nanoph-2023-0732

**Published:** 2024-01-25

**Authors:** Jiachi Ye, Chen Shen, Nicola Peserico, Jiawei Meng, Xiaoxuan Ma, Behrouz Movahhed Nouri, Cosmin-Constantin Popescu, Juejun Hu, Haoyan Kang, Hao Wang, Tarek El-Ghazawi, Hamed Dalir, Volker J. Sorger

**Affiliations:** Department of Electrical and Computer Engineering, University of Florida, Gainesville, FL 32611, USA; Department of Electrical and Computer Engineering, George Washington University, Washington, DC 20052, USA; Florida Semiconductor Institute, University of Florida, Gainesville, FL 32611, USA; Department of Material Science and Engineering, Massachusetts Institute of Technology, Cambridge, MA, USA

**Keywords:** ASPIC, PDE, analog solver, PIC

## Abstract

Solving mathematical equations faster and more efficiently has been a Holy Grail for centuries for scientists and engineers across all disciplines. While electronic digital circuits have revolutionized equation solving in recent decades, it has become apparent that performance gains from brute-force approaches of compute-solvers are quickly saturating over time. Instead, paradigms that leverage the universes’ natural tendency to minimize a system’s free energy, such as annealers or Ising Machines, are being sought after due to favorable complexity scaling. Here, we introduce a programmable analog solver leveraging the formal mathematical equivalence between Maxwell’s equations and photonic circuitry. It features a mesh network of nanophotonic beams to find solutions to partial differential equations. As an example, we designed, fabricated, and demonstrated a novel application-specific photonic integrated circuit comprised of electro-optically reconfigurable nodes and experimentally validated 90 % accuracy with respect to a commercial solver. Finally, we tested this photonic integrated chip performance by simulating thermal diffusion on a spacecraft’s heat shield during re-entry to a planet’s atmosphere. The programmable light-circuitry presented herein offers a facile route for solving complex problems and thus will have profound potential applications across many scientific and engineering fields.

## 1 Introduction

In the mathematical field, equation solving has been a key action to progress in the evolution of the human species. As a mathematical problem, partial differential equations (PDEs) are a widely known way to describe various problems, physical laws, and even economic behaviors [1]. There are examples where PDEs are used in models to describe behaviors and laws precisely, and their rapid solution is of significant practical importance. In a few instances, models that use PDEs can have precise closed-form solutions easily computed using mathematical formulas (e.g., propagation of an electromagnetic plane wave in a uniform medium). However, for most problems, close solutions cannot be found, leaving numerical solutions as the only way to achieve approximate results (e.g., for many models in fluid dynamics models that rely on Navier–Stokes equations [2]). First, digital electronics and then computers have turned numerical solving tasks into a simpler paradigm, exploiting digital computation and algorithms to achieve the searched results. Alternatively, the analog approach has existed for thousands of years, from mechanic ones, such as the sextant, to electronic analog solvers.

The interest in analog solvers within the computational landscape is rooted in their distinctive advantages over traditional digital servers. Analog computing, characterized by its continuous data processing capability, offers a more efficient approach to simulating complex physical systems, notably in solving PDEs. This efficiency stems from analog solvers’ ability to operate with natural parallelism and lower energy consumption, a stark contrast to the discrete and often energy-intensive processes of digital servers [3], [4]. Additionally, the speed of analog computation is significantly higher, reducing the latency in processing large-scale, complex computations. This makes analog solvers particularly valuable in applications demanding rapid, high-precision problem-solving. Nowadays, analog solvers mimic complex physical models and obtain solutions in a fraction of the time required by digital solvers; thus, it has great potential to solve differential equations and other mathematical problems, like using metamaterial to perform Green’s function or using metasurface to achieve paralleled computing [5]. In fact, quantum solutions such as quantum annealer like D-wave or Coherent Ising Machine, which can solve NP-hard problems in polynomial time by relying on their quantum mechanisms properties [6], [7], can be seen as analog processor empowered by quantum, although their sizes are still bulky about an integrated chip.

Photonic integrated circuit (PIC) containing optical components, such as waveguides, couplers, and modulators, that are all integrated into a compact-size chip. In recent years, PICs have raised as analog solvers and processor units, leveraging the high level of integration using CMOS fabs, the large bandwidth achievable, and the ultra-short latency. Here, we present a tunable-weight Application-Specific Photonic Integrated Circuit (ASPIC), which offers a state-of-art option for solving PDEs. By using photonics, we can take advantage of the huge bandwidth as well as the extremely low latency and power consumption to mimic the PDE models and obtain approximate results, with accuracy that reaches up to 93 % as a benchmark by commercial digital solvers.

**Figure 1:**
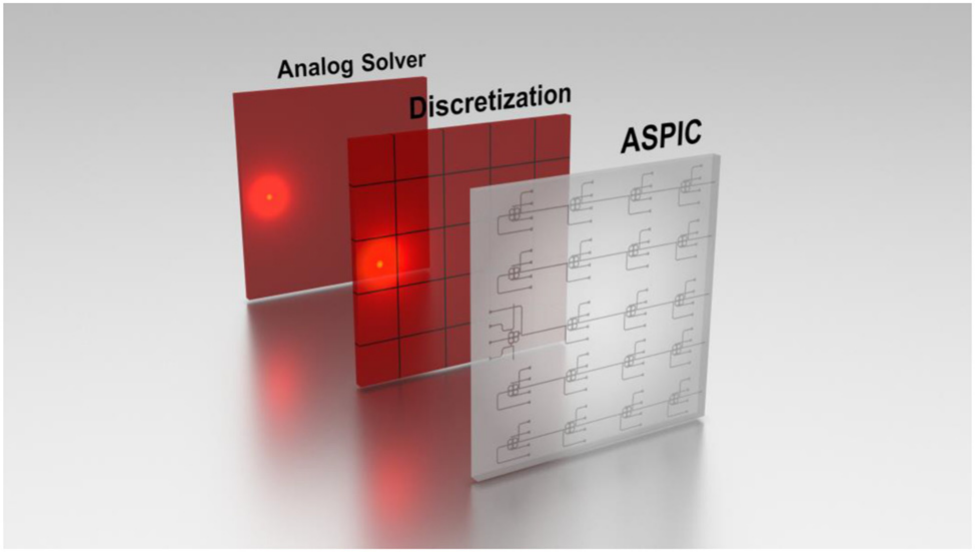
Mapping from an analytic solution of PDEs to the photonic solver.

In this paper, we first describe the photonic integrated circuits, the Kirchhoff’s Photonic Nodes as central pillar of our circuit, and the heat diffusion problems we will solve using it. Compared to the previous works [8], we show the full potential of our approach using a larger network, in a fully reconfigurable fashion, to further prove the usefulness of this circuit. We follow by experimental results of the obtained ASPIC: we will show passive and active ASPIC networks that mimic the diffusion of heat into homogeneous (passive network) and nonhomogeneous (active network) media, highlighting the high accuracy attainable by our solution. The last part is dedicated to a discussion of future improvements (Figure 1).

## 2 Methods

### 2.1 PDE-solver PIC

Several configurations can be used to match the light distribution in a PIC with the searched results of a PDE-based problem. In our case, we leverage a novel component, called Kirchhoff’s Photonic Nodes (KPNs), that we presented in the previous paper [8]. The concept behind this component is to redistribute the light as equally as possible among the different outputs, regardless of the input port. This component allows to emulate the current Kirchhoff’s law in the optical domain, under certain conditions, and so to induce a homomorphism of the circuit behavior that allows to reach an approximate solution of the PDEs model once an array of KPNs are connected together following the conditions of the problem. In our case, we will address a heat diffusion problem over a squared material, using a 5 × 5 discretization, and so a same size matrix of KPNs.

It is worth notice that the KPNs cannot perform an equal 1:3 splitting ratio [9], but the component has been designed and optimized to (i) balance the splitting ratio among the three outputs, (ii) reduce the backscatter to the input port, and (iii) limits the effects of the fabrication variability. To reach all of them, we use a symmetrical node with 3-way directional couplers and tear-shaped no-tuned microring resonators connecting the branches. This node element mimics Kirchoff’s current law in optics while inheriting all the advantages that optics brings, such as high speed, low latency, and robustness to electromagnetic interference. More details on the KPNs and the experimental output are provided in the Results section.

### 2.2 Device fabrication

The passive silicon photonic circuits are fabricated using an electron-beam lithography (EBL) process on a silicon-on-insulator (SOI) wafer by Applied Nanotools Inc. The complete circuit was designed using Klayout, a design framework for integrated circuitry. For the fabrication of the phase-change material (PCM), we deposited a GSSe (Ge_2_Sb_2_Se_5_) strip with 40 nm thickness and 100 μm length on top of the silicon waveguide, covered with a 10 nm Al_2_O_3_ layer to prevent oxidation of GSSe. Then, a 350 nm thick tungsten–titanium (W/Ti) alloy heater with identical length is paralleled, laying 1 μm away from the waveguide, routing to two 100 μm × 100 μm contact pads. On top of the contact pads and route, we deposit a 200 nm thick aluminum layer to minimize the resistance. After fabrication, we measured each heater, resulting in an 11 kΩ resistance. The base pressure before deposition was 1.7⋅10^−6^ Torr while the maximum approximate pressure during deposition was around 6⋅10^−6^ Torr. The sample was presoaked for 4 min at 6.3 % power and the deposition was done with a power ranging from 7 to 10 %. The as-deposited amorphous sample was around 39 nm thick based on a Tauc–Lorentz fit in spectroscopic ellipsometry. The parameters of the fit were approximately *A* = 193 eV, *E*_0_ = 3.51 eV, *C* = 5 eV, *E*_*g*_ = 1.69 eV, and *ϵ*_∞_ = 0. The lift-off was done overnight in acetone and the sample was subsequently rinsed with isopropanol and dried with N_2_. Approximately 10 nm of Al_2_O_3_ was deposited at 150 °C via ALD in a lab-built system similar to Anric AT-4 on the as-deposited amorphous sample for preventing oxidation.

### 2.3 Measurement setup

For the optical measurement, we used the Single-Die Probe Station as well as the measuring system from Maple Leaf Photonics. For optical power transferred outside the ASPIC, we used two 8-parallel-port fiber arrays with 127 μm pitch to inject the light in and capture the light out through grating couplers. The laser source and detector devices we use are Keysight N7778C and N7745A, respectively, both functionally programmed in the Maple Leaf Photonics measuring system. In the active measurement part, we use the National Instrument PXIe-5413 waveform generator module to generate the pulse for heating the PCM strips.

### 2.4 Heat transfer PDEs

The heat transfer simulation mapping with the partial differential equation is performed using a two-dimensional finite element model in COMSOL Multiphysics. We use the stationary “heat transfer in solid” module, which elucidates the thermal boundary conditions. The simulated area is 5 m × 5 m, equally divided into 25 (5 × 5) squares using line segments. The equations applied are:

(1) $$d_{z}\rho C_{p}u\cdot \nabla T+\nabla \cdot q=d_{z}Q+q_{0}+d_{z}Q_{\text{ted}}$$

(2) $$q=-d_{z}k\nabla T$$

where *ρ* represents the density of the material (kg/m^3^), *Q* is the heat source, *u* is the velocity, *q* is the heat flux, and *k* is the thermal conductivity (W(m⋅K)). In our stationary case, *d*_*z*_ is out-of-plane thickness, *C*_*p*_ represents the specific heat capacity (J/(kg⋅K)), and *Q*_ted_ is the thermoelastic damping.

The initial value of the whole domain is 273 K, and the heat source temperature is 393 K, while all the remaining edges are set to have a heat flux toward the outside set at 293 K to represent the heat sink of this model.

### 2.5 Optical simulation

We use Lumerical Interconnect simulation software to emulate the photonic circuit on an integrated chip. S-parameter elements with user-defined files are employed to represent the Kirchhoff Photonic Node. The continuous-wave laser is placed to generate the light at a 1550 nm wavelength. Different S-parameter elements with simple parameter numbers are exploited to mimic the PCM’s optical absorption change tuning function. We also set the splitting ratio of optical waveguide coupler elements to characterize directional couplers. Oscilloscope elements are used to read and record the power output. After 100 ps, the power fluctuation stops and a stable result can be read.

## 3 Results

In this section, a heat distribution problem over a homogeneous medium using our ASPIC will be shown. This PDE-based model can be configured with different parameters and boundary conditions, such as different thermal conductivity of the materials or the heating flux at the boundary of our model.

### 3.1 Kirchhoff Photonic Node using silicon photonics

**Figure 2:**
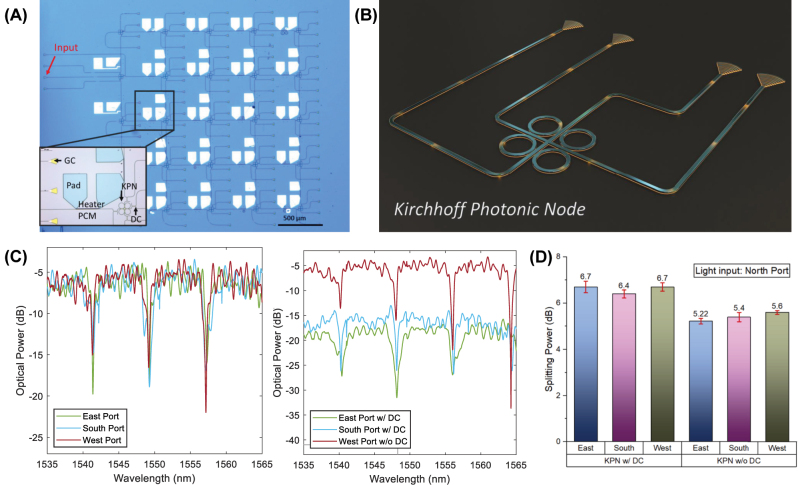
Kirchhoff Photonic Node and its matrix arrangements. (A) The ASPIC image is made by a matrix arrangement of KPNs. (B) Schematic of a Kirchhoff Photonic Node. To measure the splitting ratio, we inject the light into one grating coupler and transmit it through one waveguide. The other three grating couplers connecting to three ports will couple the light vertically out for readout. (C) Two spectra show the KPN’s port: one where ports are connected to grating couplers and the other with direction couplers that divide 10 % of the light for measurement. (D) These columns with red error bars in this chart indicate that the splitting ratio will not change with the directional coupler implementation.

A series of KPNs and a full 5 × 5 matrix were realized on a silicon photonics passive platform [10] (Figure 2A and B). We chose 500 nm waveguide width, using periodic grating couplers [11] to couple the light from the light source to the chip, as well as from the chip to the photodetectors. Similar to the electrical counterpart, we need to extract the total power from all the nodes present in the network. To do so, while reducing the losses that a direct detection would introduce, we place a 90:10 directional coupler (DC) at the output of all the node’s ports, directing the smaller amount of light to a grating coupler. By doing so, we can measure and compute the power in the node by summing the power coming from all the ports, while the larger part of the light signal will continue toward the next node. The design of the DC was performed following Lu et al. method [12]. We fabricate several different KPNs with different DC configurations that reflect the ones in the main matrix. For example, a KPN placed on the corner of the matrix will have just two DC, as the ports not facing another node do not require a splitter before the grating coupler.

Figure 2C show the spectra of two KPNs, where light is coupled from the north port. The general spectrum of the KPN shows a response resembling that of a ring resonator coming from the structure of the used KPN. In particular, KPN responses have a free spectrum range of 8 nm (998 GHz) at 1550 nm, an average bandwidth of 1.26 nm, resulting in a *Q*-factor of 1000. This component can reach a high extinction ratio of more than 15 dB. The response of the KPN is indeed an equal splitting of the power among the three outputs, which also happens partially in the resonance. However, for our purpose, we are interested in the pass-band section of the response, and this allows to compensate for any fabrication variation of the microrings without the need for tuning any microrings.

The effect of the DCs is clearly visible in the second spectrum, where East and South ports have a 10 dB reduction compared to the West. To notice that, as we consider the DC presence or absence among a series of nine different configurations of KPN, we statistically see a small difference, as shown in Figure 2D. Even in the case of having DCs, the errors due to fabrication variability are reasonably contained.

### 3.2 Silicon photonics ASPIC

Using the defined KPN, we design and fabricate a 5 × 5 passive silicon photonic matrix, as shown in Figure 2A. We placed each node 500 μm apart, linking them with a straight silicon waveguide, having a 2.1 dB/cm estimated propagation loss. The input of our matrix is placed in the first column-second row. The matrix we implement is a larger version of the previous one (from [8]), allowing a better resolution of the model we solve. Although a larger matrix is a possible option, propagation losses, as well as optical power dynamic range, must be taken into account. For example, considering using the commercial laser with a power output of 10 dBm and photodetectors with a sensitivity of −70 dBm, we can compute that the largest matrix the approach could afford will be a 6 × 6 one considering node splitting, as well as propagation and coupling losses. Some strategies to overcome this limitation will be discussed in the later section.

With this circuit, we are addressing a subset of PDEs describing heat distribution within a uniform medium. In particular, we emulate the heat distribution (Laplace Equations) over a uniform surface, having a heat source located at the input of our 5 × 5 matrix and heat flux at the boundary of the surface. We obtain our benchmark model result from COMSOL multiphysics, a commercial numerical iterative solver. Using the COMSOL solution, we can benchmark the accuracy of the solutions of a Laplace homogeneous PDE obtained by our ASPIC. The COMSOL model is formed by a 25 m^2^ square film made of a fixed material (whose heat conductivity is set). A fixed temperature point is applied at the left top of the geometry, applying a constant temperature (393 K) as a heat source. Simultaneously, all the remaining edges are set to have a heat flux toward the outside, which is set at 293 K to represent the heat sink of this model. We set the computation domain to be divided into 25 smaller squares, represented by our 5 × 5 model, and computed the average temperatures of all these squares. This model can simulate the thermal transfer profile across the domain under the Dirichlet boundary conditions.

**Figure 3:**
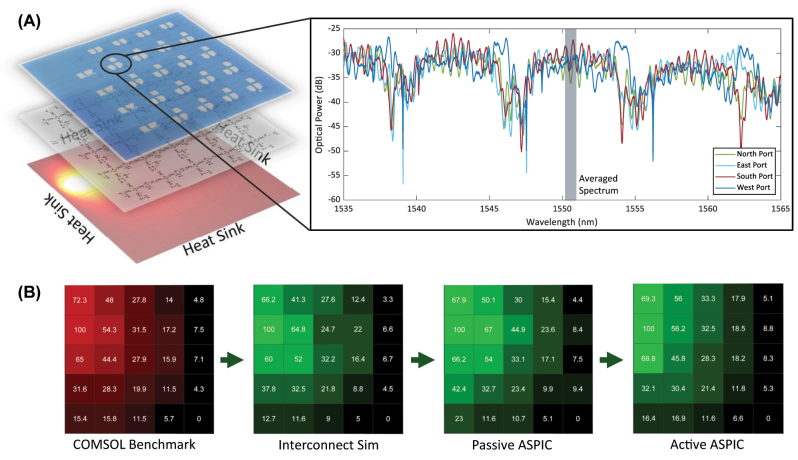
ASPIC PDE solver. (A) The stacking diagrams are the correlation of different solver’s power maps from the COMSOL solver to interconnect solver and ASPIC solver. The analysis figure along the diagrams shows the power readout spectrum of one KPN in the ASPIC, indicating that the power splitting of four ports is analogous. (B) These power maps demonstrate the thermal distribution in COMSOL solution and optical power distribution in interconnect and ASPIC solver. From the accuracy chart, we can see that both accuracy of interconnect and passive ASPIC cannot reach above 90 %.

Besides our experimental ASPIC, we built a one-on-one 5 × 5 optical mesh on the Lumerical Interconnect software to simulate the ASPIC and imitate the heat transfer mesh structure. At the crossing nodes, we used the scattering matrix parameter (S-parameter) of the KPN, computed to reproduce its optical characteristics. Similar to our silicon photonic ASPIC, we extract the power from each node by placing a DC in all the ports of the KPN. Moreover, all the nodes are connected by a waveguide model, whose propagation loss is set to the experimental value and tuned to match the heat conductivity of our model. The three models (Comsol, Lumerical, experimental) are shown in Figure 3A.

#### 3.2.1 Passive ASPIC results

We start by measuring the passive version of our ASPIC, whose propagation losses between nodes are not tuned. We inject a 10 mW tunable continuous-wave laser, centered at 1550 nm, through the grating coupler at the input node and then collect the power output from all the nodes’ ports to compute the actual power for our model. A spectrum of the four outputs from one KPN is shown in Figure 3A. The spectra are similar to the one presented in the previous section, with the same FSR. However, in this case, we can notice that the notches do not have the same shape or bandwidth as those of a single KPN. Since this node is inside a matrix, its spectrum results from all the incoming light beams from adjacent nodes that have slightly de-tuned microring notches, resulting in a more complex line shape. Moreover, the nonoptimized grating couplers produce a Fabry–Perot ripple that we eliminate by averaging over one period, shown in Figure 3B, equivalent to have a large bandwidth laser at the input.

After collecting the data from both the measurement and the simulations, considering that those quantities are incoherent in terms of measured units, we opted for a normalization scheme (0–100) between the minimum and maximum quantity obtained. The data are shown in Figure 3B, as a heat map of COMSOL, Interconnect simulation, and experimental data from the passive ASPIC.

From these results, we can define the accuracy as:

(3) $$A=1-\overset{n}{\underset{i=1}{\sum }}\frac{\left|N_{i,c}-N_{i,a}\right|}{N_{i,c}},\;$$

where *A* is the total accuracy of the solution, *n* is the number of the nodes, *N*_*i*,*c*_ and *N*_*i*,*a*_ are, respectively, the node values of COMSOL solution and solver solution (simulated or measured), normalized to a 0–100 % range. With our model, we obtain an 89.5 % accuracy for the Interconnect solution and an 86.4 % accuracy for the experimental ASPIC.

The obtained accuracy is slightly below 90 %, mainly due to the mismatch of optical properties over the matrix, as well as the passive structure that does not allow tuning the losses between nodes to match the physical heat properties of the material in the model. However, the ASPIC solver can compute the model with a latency of hundreds of ps (time for the light to propagate over the entire circuit), compared to the COMSOL model, which requires several ms, making the optical solution several orders of magnitude faster.

#### 3.2.2 Active ASPIC results

To further improve the accuracy of our ASPIC, a tunable element has to be placed between each node pair to increase the losses and match any material properties. Among all the components we can use for this scope, such as MZI, variable-optical attenuator, and so on, we chose a nonvolatile tunable element based on an integrated phase change material (PCM), whose optical properties can be modulated with its crystalline structure. This choice enables ultra-compact integration, reduced system complexity, and high energy efficiency taking advantage of the nonvolatile nature of PCM. Among the many PCM materials, we select GSSe (Ge_2_Sb_2_Se_5_) since it provides an excellent extinction ratio between its amorphous and crystalline states while having an almost negligible insertion loss at the C-band.

##### 3.2.2.1 Phase change materials for tunable component

**Figure 4:**
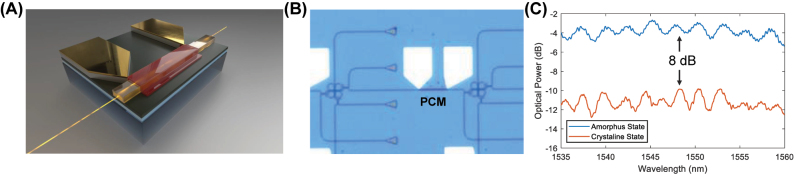
PCM implemented full active ASPIC solver. (A) 3-D schematic diagram of PCM and heater design, patterning using electron-beam lithography and sputter deposition for dual metal layers. (B) The optical image of the heater and contact pad is shown in panel A. (C) This plot shows the spectrum of waveguide power detected with contrast states of the PCM strip employed. By applying a hybrid pulse to the heater, the PCM switched from an amorphous state to a crystalline state, increasing the extinction ratio by 0.08 dB/μm.

The integration of the GSSe has been done by depositing a thin strip over the waveguide and implementing a heater scheme to provide the Joule heating energy to switch the material between its two states [13]. A graphical representation of the implementation is shown in Figure 4A. We place this component between each KPN node, as shown in Figure 4B, permitting full reconfigurability of the ASPIC.

A heuristic approach is taken to study the sufficient power required for switching the GSSe’s state. In accordance with the measurement results, we observed a hybrid pulse comprised of a 12 V high voltage for 500 μs and a 9.6 V low voltage for 2 ms can switch the GSSe strip from the amorphous state to the crystalline state (Figure 4C). The spectrum plotted indicates that the absorption is 8 dB, which implies a 0.08 dB/μm absorption coefficient of the GSSe on-chip. Significantly, the imaginary part of the effective refractive index in the amorphous state is 2.18 × 10^−5^, leading to an exceedingly small unit of passive insertion loss [14].

##### 3.2.2.2 Active ASPIC using PCM

**Figure 5:**
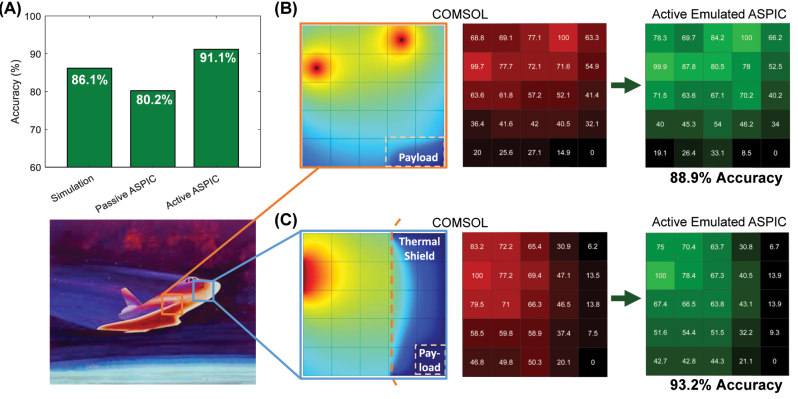
(A) Comparison between the COMSOL heatmap and the power map obtained by the active ASPIC. In this case, the ASPIC can solve PDEs with a stable accuracy of over 91 %. (B) and (C) Two heat models for different spacecraft cases including one or two heat sources and heatshield. The heatmap and power maps show the modeling of the thermal shield that is protecting the payload under the re-entry phase of a space journey. Even in these demanding cases, the accuracy of our active ASPIC remains higher than 88.9 %.

The tunable PCM elements implemented in our circuit impart full programmability to the ASPIC. Following a heuristic approach of switching different states in single or multiple GSSe strips within the matrix, we monitor the accuracy variation and seek the highest accuracy value. Over the course of time, by selectively switching the GSSe strips from the amorphous state to the crystalline state, we successfully achieved the solution corresponding to the same problem, solved by the Interconnect solver and passive ASPIC solver, with an accuracy of 93.2 % (Figure 3B), increased by 7.8 % from the full passive ASPIC (Figure 5A).

To demonstrate the flexibility of our active ASPIC, we propose two more approximate models of heat shields present in all space vehicles [15]. In both cases, this shield has to protect the whole vehicle against high-temperature environments in different parts of the vehicle body, particularly during the re-entry phase into the atmosphere, when the vehicle gets exposed to temperatures of more than 1000 °C [16]. In the first case, we emulated a thermal shield with low heat conductivity in dual high-temperature sources situation that has the main goal to shield the bottom right square from the higher temperature source in Figure 5B. After obtaining the COMSOL result as a benchmark, we iteratively switched various PCM strips between different nodes in the Lumerical emulation of the active ASPIC following the same heuristic approach used before. Thus, we can program the ASPIC to adopt the new model with 88.9 % accuracy, as shown in the latter heatmaps of Figure 5B. Similarly, the second COMSOL model presents a high-temperature source and a thermal shield. The first heatmap in Figure 5C shows the actual temperature distribution and the strong thermal insulation provided by the heat shield, and the accuracy of this model is 93.2 %. We can also observe from the power map comparison that the highest error occurs at the first layer in the heat shield. The explanation is that our PCM “modulators” only have a fixed tunable extinction ratio of 8 dB, so we cannot arbitrarily tune the extinction ratio precisely in various states. However, by means of applying more PCM strips with different lengths deposited on the waveguides, we will be allowed to address this problem and increase the accuracy.

## 4 Discussion

As we showed, it is possible to solve the PDE model using photonic integrated circuits, achieving high accuracy. In particular, the analog photonic approach to solves the PDEs model allows to reach the results ideally ps-level, once correct reading components are placed, compared to several clock cycles that are needed for digital processors. This orders-of-magnitude improvement permits to reduce the amount of energy consumption to pJ-level, as the only power consumption is the laser and the photodetectors reading for the time needed. However, the circuit shows some limitations that must be taken into account.

First of all, the separation between two adjacent nodes sets the minimum amount of loss (as propagation loss) that the ASPIC has. This reflects a boundary in the model we want to solve, as the heating material cannot have a heat conductivity larger than the equivalent value obtained from the photonic circuit. This power budget problem can be addressed in several ways, from using a low-loss waveguide (large-core or ridge silicon waveguides, or silicon nitride [SiN] waveguides) for reducing the distance between nodes.

In the case we showed in this paper, the ASPIC can solve the Laplace Equation with over 90 % accuracy, which is a number that is worth to be discussed among the analog solvers. In a published paper that uses an analog solver to accelerate a digital solver for solving Burger’s equation, the accuracy is 94.62 % [17]. Another work using a large programmable VLSI analog computer to solve heat equations reached 97.4 % accuracy but using a bulky device [18]. In another paper, researchers used a single chip to solve the 1-D wave equation with a 2 % error [19]. The accuracy of these solving mathematical problems with analog solvers cases is 90-ish percent, a range where also our ASPIC is placed. Compared with these works, the ASPIC has a slightly lower accuracy, but it has a higher speed, low latency, and a more compact size.

In fact, this latter one comes with the need to assess the optical power out from every single node. As shown in Figure 4B, most of the space is occupied by the grating couples and directional couplers from each node’s output ports. This design has several limitations, such as footprint occupation and power budget, since it requires 90:10 couplers and low cross-talking cross-nodes. Using integrated photodetectors potentially improves the overall KPN footprint. Solutions, such as CLIPP [20], [21], will further enhance this design, as they have the feature of being transparent, and they can be directly integrated into the waveguide to be monitored. The current ASPIC has a total spatial footprint of the circuit is around 3 mm × 3 mm, mainly due to the 500 μm of space between each KPN occupied by the metal pads. Possible solutions to reduce the footprint can come from the technology side, as well as the packaging. Nowadays, chip manufacturers offer up to 14 metal layers on the Back-End-Of-Line (e.g., GlobalFoundries 45SPLCO process [22]), allowing for a better and more compact metal routing so that the electronic bond-pads won’t be a limiting scaling factor for the KPN node density. Moreover, the package of the chip can be improved by using through-via technology, for example, reducing the space between nodes to only 50 μm (one KPN size). With all these improvements, even a finer mesh grid size of 1000 × 1000 would have the dimension limited to 5 cm × 5 cm. An optimized inverse design approach can further reduce the size, by which the design area can be shrunk to 2 μm dimension, but it will require a completely different configuration and related optimization. Larger meshes will be limited by the propagation loss, as the light output at the furthest node will be below the detectable range of the current photodetector technology.

Kirchhoff’s law is widely used in analog electrical mesh processors, for solving second-order partial differential equations applying finite difference methods. The key core of the KPN realizes Kirchhoff’s law in the optical domain, so, we intend to apply the ASPIC to partial differential equations. However, for solving nonlinear differential equations like the Navier–Stokes equation, more nonlinear photonics components like WGM microresonance devices or EDFA with self-phase-modulation are needed in the circuit [23], which will significantly increase the fabrication complexity and power consumption. Thus, we limit the aim of the current ASPIC to linear differential equations.

The last point focuses on boundary conditions. In our circuit, every port toward the outside of the matrix is directly connected to a grating coupler for coupling all the light out. This can be seen in the heat model as a heat flux toward the outside. However, to fully generalize our ASPIC to all the possible models, we have to permit different configurations at each external port, for example, switching between a grating coupler, which extracts all the optical signal and a Sagnac Loop [24], whose function is to reflect the light completely back into the matrix. This later solution could be used as heat insulation, providing an ASPIC that can solve more complex PDE models [25].

In summary, we design and test an Application-Specific Photonic Integrated Circuit using KPN capable of solving the analog partial differential equation for a heat distribution problem with over 90 % accuracy. The silicon photonics integration permits obtaining a chip with a size smaller than a fingernail, over 50 nm optical bandwidth, and most importantly, obtaining the results on a nanosecond scale. We show a passive and active version of the ASPIC, showing the higher accuracy we can achieve once we can tune the phase change material between two nodes to match the properties of the model materials. With the progressive Through Silicon Via and multi-layer technology [26], we will be able to design the optical and electrical routes through different layers, achieving more complicated functionalities. Also, by adding the ring modulators to the matrix, it is possible to reconfigure each node to achieve different splitting ratios, so that different types of circuits can be configured, from optical filter, multiplexer, de-multiplexer, and more complicated versions for artificial intelligence and neuromorphic computing [27].
